# Aberrant DNMT1-mediated DACH1 methylation is associated with colorectal adenoma-to-carcinoma progression

**DOI:** 10.3389/ebm.2025.10469

**Published:** 2025-04-30

**Authors:** Yan Zhang, Honggang Liu

**Affiliations:** ^1^ Department of Pathology, Beijing Tongren Hospital, Capital Medical University, Beijing, China; ^2^ Department of Pathology, Beijing Changping Traditional Chinese Medicine Hospital, Beijing, China

**Keywords:** DNMT1, DACH1, colorectal cancer, promoter methylation, tumor progression

## Abstract

Colorectal cancer (CRC) remains a major contributor to cancer-related morbidity and mortality. While Dachshund homolog 1 (DACH1) was recognized as a critical regulator in cancer progression, its role in promoting or suppressing tumor development remains a subject of ongoing debate. This study aimed to elucidate the role of DACH1 in CRC progression and its underlying regulation mechanisms. The expression levels of Methyltransferase 1 (DNMT1) and DACH1, as well as its methylation status were assessed through a combination of TCGA data analysis and experimental validation using immunohistochemistry, PCR, methylation-specific PCR, and bisulfite sequencing RCR on 120 clinical samples, comprising normal mucosa, adenomas, and adenocarcinomas. The relationships among them were evaluated using Pearson or Spearman correlation analysis. The associations between the DACH1 and DNMT1 levels and clinicopathological parameters were examined to determine their clinical relevance. A progressive decrease in DACH1 expression and a concomitant increase in DACH1 promoter methylation and DNMT1 expression were observed from normal mucosa to adenoma and adenocarcinoma tissues. Higher DNMT1 expression and lower DACH1 expression were associated with poorer clinical outcomes, including worse tumor differentiation, lymphatic metastasis, and advanced tumor stages. Paired analysis of tissues from the same patient further validated their inverse expression patterns during CRC progression. DNMT1-mediated DACH1 epigenetic silencing plays a critical role in CRC progression, suggesting that the DNMT1-DACH1 regulatory axis may serve as a potential biomarker and therapeutic target in CRC.

## Impact statement

The present study revealed a crucial epigenetic mechanism in colorectal cancer (CRC) progression involving the aberrant regulation of DACH1 by DNMT1 based on the combination of clinical sample analysis with bioinformatic databases. We demonstrated that DACH1 expression progressively decreased from normal colorectal mucosa to adenoma and adenocarcinoma tissues, while its methylation level and DNMT1 expression exhibited the opposite trend. Clinical parameters further highlighted the association of DACH1 and DNMT1 expression with tumor differentiation, lymphatic metastasis, and tumor stage. Our findings suggested that DNMT1-mediated hypermethylation might contribute to the silencing of DACH1, thereby promoting CRC progression. This provided a novel perspective into the epigenetic regulation of tumor suppressor genes in CRC and identified potential therapeutic targets for intervention.

## Introduction

Colorectal cancer (CRC) is one of the most commonly diagnosed cancer worldwide and is projected to increase to 3.2 million new cases annually by 2040, with a mortality rate exceeding 50% [1]. This alarming trend underscores its significant threat to global public health. The progression of CRC typically follows a well-established “normal mucosa-adenoma-carcinoma” sequence, which is driven by the dysregulation of tumor suppressor genes and oncogenes, leading to the initiation and progression of malignancy [2]. Under normal physiological conditions, colorectal epithelial cells maintain a delicate balance between proliferation and apoptosis, ensuring mucosal tissue stability. However, disruptions in this balance create a permissive environment for tumor initiation, which is modulated by some key genes [1].

The development of adenomas marks a critical early event in CRC pathogenesis, as adenomas are recognized as precancerous lesions that can progress to carcinomas. Mutations in key genes such as APC and KRAS are frequently observed during this stage [3, 4]. As adenomas progress, tumor cells may acquire additional mutations and accumulate epigenetic changes, facilitating the transition to invasive carcinoma. For instance, the inactivation of the TP53 gene disrupts cell cycle regulation, thereby promoting the development of carcinoma *in situ* [5]. Understanding the molecular mechanisms underlying this progression is essential for the development of effective diagnostic and therapeutic strategies to combat CRC.

Emerging evidence has identified DACH1 as a tumor suppressor gene involved in regulating key processes such as cell proliferation, apoptosis, and invasion across various cancers including breast, prostate, and lung cancers [6–8]. For example, the deletion of the DACH1 gene occurs in up to 18% of human prostate cancer cases, and its loss in mouse prostate cancer models has been shown to enhance prostatic intraepithelial neoplasia and DNA damage [6]. In CRC, previous studies suggest that the loss of DACH1 expression is associated with tumor progression [9, 10]. However, conflicting evidence suggests that DACH1 is highly expressed at all stages of CRC, and increased DACH1 expression was identified as an independent predictor of poor prognosis, highlighting its pro-tumorigenic role through the regulation of BMP signaling [11]. Based on this conflicting evidence, the specific role and regulatory mechanisms of DACH1 expression in CRC require further investigation.

Gene expression during tumor progression is regulated through a variety of mechanisms, among which epigenetic modifications, particularly promoter region DNA methylation, play a pivotal role in regulating gene expression without altering the underlying DNA sequence [12]. Aberrant hypermethylation of promoter CpG islands is a common event in cancer, leading to the silencing of tumor suppressor genes [13]. In CRC, for example, the promoter hypermethylation of PRDM2 and MLH1 is initiated in sessile serrated lesions, which can progress into MLH1-deficient CRC [13]. Additionally, DNA methylation homeostasis is closely related to DNA Methyltransferase 1 (DNMT1), which is a hallmark of various malignancies [14, 15]. Despite overexpression of DNMT1 having correlation with progression, poor prognosis and chemoresistance in CRC [16, 17], whether it exerts this negative regulation through the methylation of DACH1 remains unclear.

As such, our study systematically analyzed the expression patterns of DACH1 and DNMT1 across normal tissues, adenomas, and adenocarcinomas by integrating clinical samples with data from the TCGA database. Our findings revealed the importance of the DNMT1-mediated epigenetic regulation of DACH1 in CRC initiation and progression and clarified its relationship with clinical parameters. Targeting this pathway might represent a promising therapeutic strategy for CRC.

## Materials and methods

### Ethical statement

The study was conducted in accordance with the Declaration of Helsinki and was approved by the Ethics Committee of Beijing Tongren Hospital. Written informed consent was obtained from all participants before sample collection. Patient information was anonymized to protect confidentiality, and all data were handled following institutional and legal guidelines for patient privacy and research ethics.

### Clinical sample collection and grouping

A total of 120 tissue samples were collected from patients who visited the Department of General Surgery at Beijing Tongren Hospital between 2016 and 2022. The samples included 40 cases each of normal colorectal mucosa, colorectal adenoma, and colorectal adenocarcinoma tissues, confirmed by pathological diagnosis. Patients had received radiotherapy or chemotherapy were excluded prior to sample collection. Clinical and pathological data, including age, gender, tumor differentiation degree, lymph node metastasis status, and tumor stage (Dukes staging), were collected and analyzed based on the expression levels of DACH1 and DNMT1.

### Criteria for tumor classification and staging

Tumor differentiation degree was evaluated based on the morphology and function of tumor cells to normal tissues. High differentiation indicates cells closely resembling normal cells, retaining partial structure and function, with lower malignancy and better prognosis. Moderate differentiation represents partially disorganized structures and intermediate malignancy. Low differentiation shows severe disorganization, higher malignancy, and worse prognosis. Lymph node metastasis was classified based on regional lymph node involvement. Dukes staging was used to evaluate CRC progression. Dukes A and B represent that tumor confined to mucosa, submucosa or invades muscularis propria without lymph node metastasis, whereas Dukes C and D with lymph node or distant metastases.

### Immunohistochemistry (IHC)

Tissue samples were fixed in 10% formalin, embedded in paraffin, and sectioned into 4 μm slices. Sections were deparaffinized with xylene, hydrated through graded ethanol, and subjected to antigen retrieval using sodium citrate buffer under high-pressure heat. Endogenous peroxidase activity was blocked by incubating sections in 3% hydrogen peroxide for 10 min, followed by blocking with 5% bovine serum albumin for 30 min. Sections were incubated overnight at 4°C with primary antibodies anti-DACH1 (1:200; Abcam, Cambridge, UK) and anti-DNMT1 (1:200; Cell Signaling Technology, Danvers, MA, United States). The following day, sections were incubated with biotinylated secondary antibodies (Cell Signaling Technology, 1:200) at room temperature for 30 min. Signal detection was performed using DAB staining, followed by hematoxylin counterstaining, dehydration, clearing, and mounting. DACH1 and DNMT1 expression was assessed independently by two pathologists using a semi-quantitative scoring system based on the proportion of stained cells (0: <5%, 1: 5%–25%, 2: 26%–50%, 3: 51%–75%, 4: >75%) and staining intensity (0: negative, 1: weak, 2: moderate, 3: strong). Total scores ranged from 0 to 7, categorized as negative (−, 0–1), weak (+, 2–3), moderate (++, 4–5), and strong (+++, 6–7).

### Bioinformatics analysis

RNA-sequencing data of DACH1 and DNMT1 from 589 CRC patients were obtained from The Cancer Genome Atlas (TCGA) database^1^, with DACH1 methylation data (cg26413827; 1st Exon, 5’UTR) derived from Illumina HM27 or HM450 platforms. RNA-sequencing data were used to analyze the mRNA expression levels of DNMT1 and DACH1, while the methylation status of the DACH1 were evaluated by calculating the average Beta values for CpG sites. To investigate the relationship between DACH1 methylation levels and the expression of DACH1 or DNMT1, samples were divided into two groups based on the median mRNA expression levels of DACH1 (low, n = 295; high, n = 294) or DNMT1 (low, n = 295; high, n = 294). The methylation levels of DACH1 were then compared between these two groups to determine any significant differences and assess potential correlations between these mRNA expressions with DACH1 methylation.

### Quantitative real-time PCR (qRT-PCR)

Total RNA was extracted from fresh-frozen tissue samples using TRIzol reagent (Invitrogen, Carlsbad, CA, United States) following the manufacturer’s instructions. RNA purity and concentration were assessed using a NanoDrop 2000 spectrophotometer (Thermo Fisher Scientific, Waltham, MA, United States). One microgram of total RNA was reverse-transcribed into cDNA using the PrimeScript™ RT reagent Kit (Takara, Shiga, Japan). qRT-PCR was performed on an ABI 7500 Real-Time PCR System (Applied Biosystems, Foster City, CA, United States) using SYBR^®^ Premix Ex Taq™ II (Takara). The primers were used to detect DACH1 (Forward: 5′-AGC​AGC​AGC​GAG​TAC​AAG​AA-3′, Reverse: 5′-CTG​CTG​CTG​TTG​CTG​TTG​TT-3′) and DNMT1 (Forward: 5′-GTG​CTG​CTG​CTG​CTG​CTG​TA-3′, Reverse: 5′-ACA​CAC​ACA​CAC​ACA​CAC​AC-3′). PCR conditions were set as: 95°C for 30 s, followed by 40 cycles of 95°C for 5 s and 60°C for 30 s. Relative gene expression was calculated using the 2^−ΔΔCT^ method, normalized to GAPDH.

### Methylation-specific PCR (MSP) and bisulfite sequencing PCR (BSP)

The genomic DNA was extracted from normal mucosa, adenoma, and adenocarcinoma tissues using a standard phenol-chloroform method. The extracted DNA was then bisulfite-converted using the EZ DNA Methylation-Gold™ Kit (Zymo Research, Irvine, CA, United States) following the manufacturer’s instructions. Methylation-specific primers for the DACH1 promoter were designed using MethPrimer^2^, targeting both methylated and unmethylated CpG sequences. PCR amplification was performed in a 25 μL reaction mixture containing 2 μL bisulfite-treated DNA, 0.2 μM primers, and 12.5 μL 2× Taq PCR Master Mix (Takara, Shiga, Japan). Thermal cycling conditions were as follows: initial denaturation at 95°C for 10 min, followed by 40 cycles of 95°C for 30 se, 58°C for or 56°C for 30 s for methylated primers and unmethylated primers, respectively, and 72°C for 30 s, with a final extension at 72°C for 10 min. The PCR products were cloned into the pGEM-T Easy vector (Promega, Madison, WI, United States) and transformed into *Escherichia coli* DH5α competent cells. Positive clones were selected and plasmid DNA was extracted using the QIAprep Spin Miniprep Kit (Qiagen, Hilden, Germany). The inserted fragments were sequenced using the Sanger sequencing method. Sequencing reactions were performed using the BigDye™ Terminator v3.1 Cycle Sequencing Kit (Thermo Fisher Scientific) following the manufacturer’s protocol. The reaction products were purified using a Sephadex™ G-50 column and analyzed on an ABI 3730xl DNA Analyzer (Applied Biosystems). Each CpG site was classified as methylated (black circles) or unmethylated (white circles), and detailed methylation patterns were visualized to create single-molecule methylation maps. The methylation rate was expressed as the ratio of methylation-positive cases.

### Luciferase reporter assay

To confirm whether DACH1 methylation regulates the transcriptional activity of its promoter, a luciferase reporter assay was performed using the luciferase reporter vector comprising the pGL3-basic vector (Promega) with the DACH1 promoter sequence containing the binding site. The above vector plasmids were transfected into 293 T cells (ATCC) using Lipofectamine 2000, respectively. After 48 h of transfection, the Dual Luciferase Reporter Gene Assay System kit (Promega) was applied to carry out luciferase assays.

### 5-Aza-2′-deoxycytidine treatment

HCT116 cells purchased from Procell (Wuhan, China) were treated with a final concentration of 10 μM of 5-aza-2′-deoxycytidine (5-Aza-dC, Sigma-Aldrich, Steinheim, Germany), which is a DNMT1 inhibitor. After 3 days, the treated cells were collected for further detections, including qRT-PCR, western blot, and MSP. The cells treated with PBS served as the control.

### Western blot

The total protein was lysed from the cells and subsequently quantified by BCA kit analysis (Thermo Fisher), separated by 10%SDS-PAGE gel electrophoresis, and transferred to PVDF membranes (Millipore Corp, Billerica, MA, United States). Next, the membranes were sealed and then incubated overnight at 4°C with the primary antibody anti-DACH1 (Abcam), and with the secondary antibody for 2 h. Finally, the protein bands were visualized by the ECL Western Blotting Substrate Kit (Abcam). Relative expression of proteins was analyzed using Quantity One software with GAPDH as an internal reference.

### Statistical analysis

All data were analyzed using SPSS 22.0 software (IBM Corp., Armonk, NY, United States). Continuous variables were expressed as Mean ± SEM and compared using one-way ANOVA or the Kruskal-Wallis test, followed by Tukey’s HSD post-hoc test. Categorical variables were analyzed using chi-square tests. Correlations between parameters were assessed using Pearson or Spearman correlation analysis, with scatterplots generated to visualize trends. Regression analysis was performed to evaluate the degree of correlation. Statistical significance was defined as p < 0.05.

## Results

### DACH1 expression progressively decreased in normal, colorectal adenoma and adenocarcinoma tissues

**FIGURE 1:**
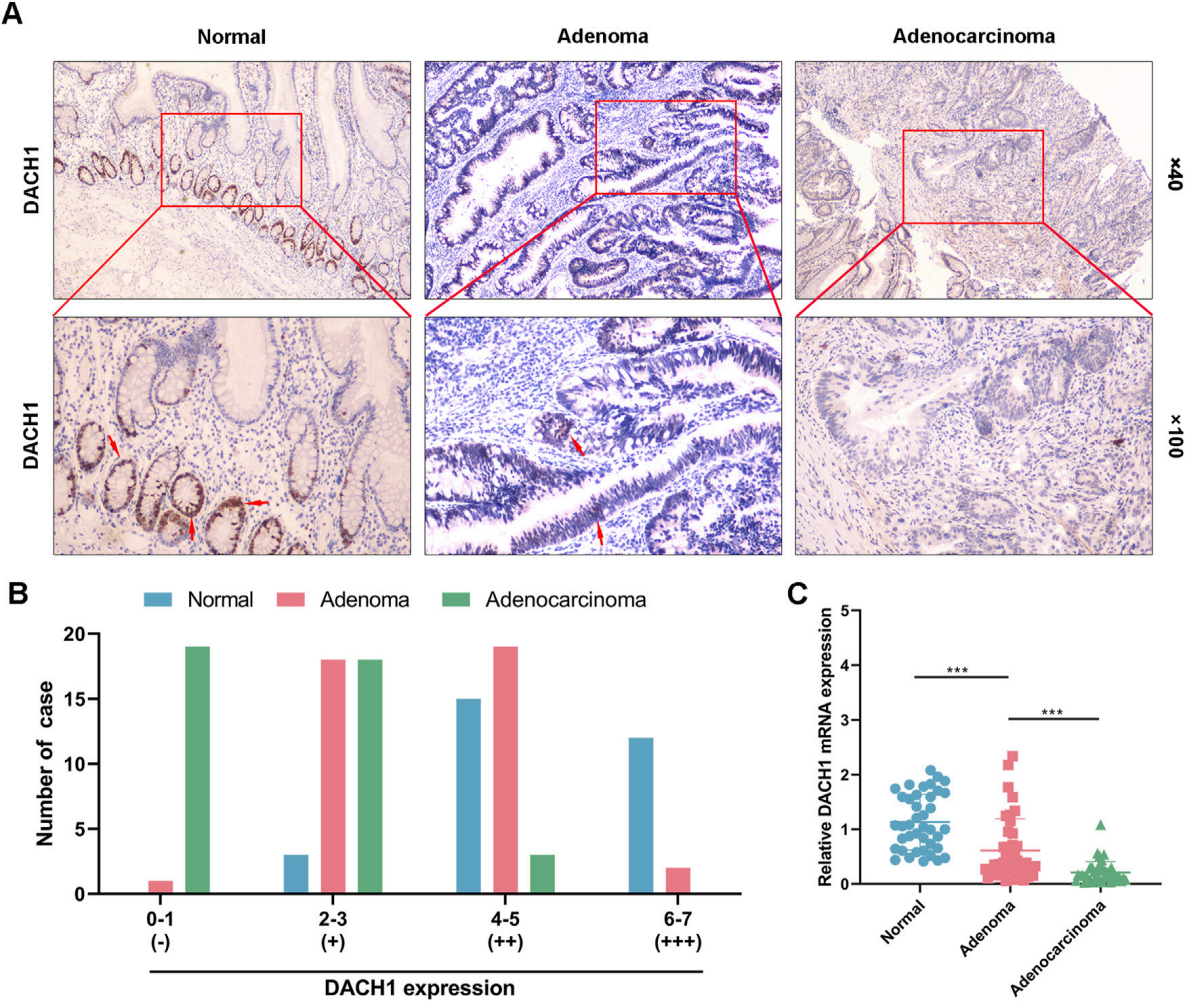
DACH1 expression in normal, colorectal adenoma, and adenocarcinoma tissues. Forty cases each of normal colorectal mucosa, colorectal adenoma, and colorectal adenocarcinoma tissues were collected from patients. **(A)** The DACH1 protein expression determined by immunohistochemical analysis in these tissues. Representative images were demonstrated by the magnification of 100 × and 400 ×. **(B)** Immunohistochemical staining intensity evaluated using semi-quantitative scoring for negative (−), weak (+), moderate (++), and high (+++) DACH1 expression across the “normal-adenoma-adenocarcinoma” progression. **(C)** Relative mRNA expression of DACH1 was measured by qRT-PCR. Data are presented as mean ± SEM. ***p < 0.001.

Forty cases of each type, including normal colorectal mucosa, colorectal adenoma, and colorectal adenocarcinoma tissues, were collected for analysis of DACH1 levels. The expression of DACH1 protein was assessed through IHC. A progressive reduction in DACH1 expression was observed from normal colorectal mucosa to adenoma and adenocarcinoma tissues. In normal tissues, DACH1 was predominantly localized in the nuclei of epithelial cells, showing strong staining intensity, whereas moderate staining was detected in adenoma tissues, and weak or negligible nuclear staining was evident in adenocarcinoma tissues (Figure 1A). Half-quantification of IHC staining scores revealed a significant decline in the number of cases with high DACH1 expression (+++) across the “normal-adenoma-adenocarcinoma” progression, with its level being negligible in the adenocarcinoma group. Conversely, cases with negative (−) staining were significantly increased in adenocarcinoma tissues compared to normal and adenoma tissues. While weak (+) staining was elevated in both adenoma and adenocarcinoma relative to normal tissues, no significant difference was observed between adenoma and adenocarcinoma cases. The number of cases with moderate (++) staining was markedly reduced in adenocarcinoma compared to both normal and adenoma tissues (Figure 1B). Additionally, the relative mRNA expression of DACH1 followed a similar trend, with the highest levels observed in normal tissues, followed by a significant decrease in adenoma and the lowest levels in adenocarcinoma tissues. Statistical analysis confirmed highly significant differences in DACH1 mRNA expression among these three groups (Figure 1C). These findings indicated a progressive loss of DACH1 expression at both the protein and mRNA levels during the “normal mucosa-adenoma-adenocarcinoma” sequence, suggesting that the downregulation of DACH1 may play a crucial role in the pathogenesis and progression of colorectal cancer.

### Promoter hypermethylation of DACH1 contributed to its transcriptional silencing in colorectal tissues

**FIGURE 2:**
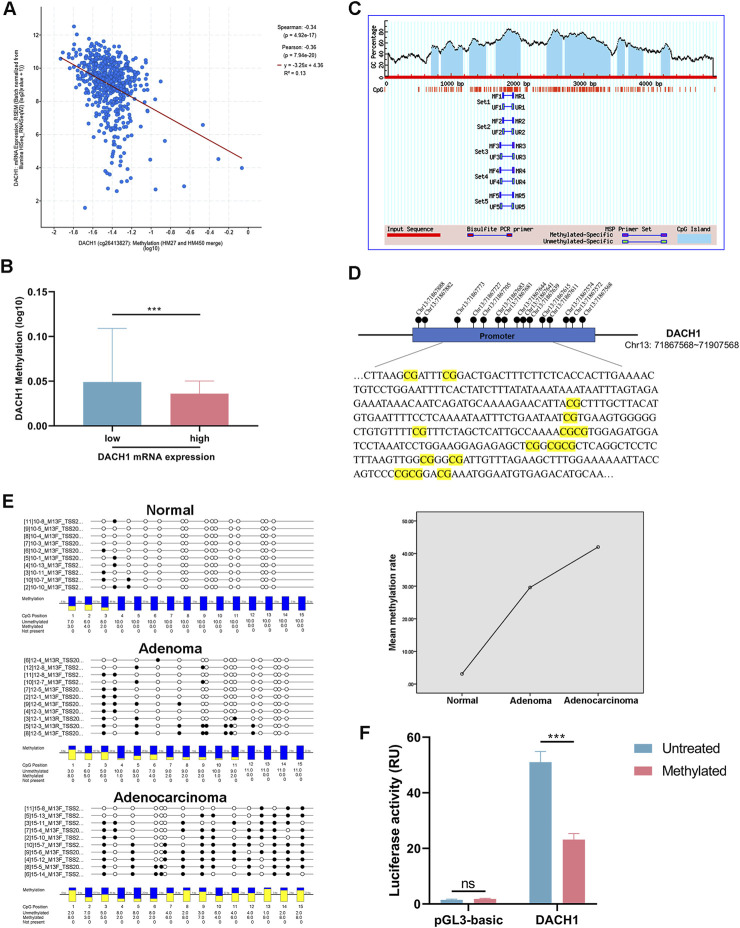
Relationship of DACH1 expression and promoter methylation cross colorectal cancer progression. **(A)** Correlation analysis of DACH1 promoter methylation levels and mRNA expression and **(B)** comparison of DACH1 methylation levels between the patients stratified by DACH1 median mRNA expression in colorectal tissues based on the TCGA database. **(C)** Genomic organization of the DACH1 promoter region and visualization of CpG island regions targeted by methylation-specific PCR. **(D)** The schematic diagram of the DACH1 promoter and the location of 15 CpG sites detected in this study. **(E)** Methylation profiling across normal mucosa, adenoma, and adenocarcinoma tissues and quantitative analysis of mean methylation rates across the three stages. **(F)** Luciferase reporter assay confirmed that DACH1 methylation regulates the transcriptional activity of its promoter. Data are presented as mean ± SEM. ***p < 0.001.

The relationship between DACH1 methylation and transcriptional silencing in colorectal cancer tissues was investigated using methylation data from TCGA (n = 589). A significant negative correlation was identified between the methylation levels of DACH1 CpG sites (cg21343827) and its mRNA expression levels (Pearson correlation coefficient: r = −0.36, p < 0.001) (Figure 2A). Using the same data, we divided the samples into two groups (low and high DACH1 mRNA expression groups) based on the median DACH1 mRNA levels. A comparison of methylation levels revealed significantly higher promoter methylation in patients with low mRNA expression compared to those with high DACH1 expression (Figure 2B). This suggested that increased methylation of the DACH1 is associated with reduced transcriptional activity. To validate these findings, MethPrimer^3^ was applied to design primer to target critical CpG island regions within the DACH1 promoter (Figure 2C). The genomic organization of the DACH1 promoter and the specific CpG regions analyzed were visualized (Figure 2D). Based on BSP analysis, detailed methylation profiling across the normal mucosa-adenoma-adenocarcinoma sequence revealed that most CpG sites within the DACH1 promoter were unmethylated in normal mucosa. Moderate increases in methylation were observed in adenoma tissues, while adenocarcinoma tissues exhibited a significant and widespread increase in methylation levels. Quantitative analysis showed a progressive increase in mean methylation rates from normal mucosa to adenoma and adenocarcinoma (Figure 2E). Moreover, the dual-luciferase reporter assay showed that methylation of the DACH1 promoter caused a significant reduction in its transcriptional activity (Figure 2F). These results demonstrate that DACH1 promoter hypermethylation is associated with transcriptional silencing, suggesting that this epigenetic modification may play a pivotal role in the adverse progression of CRC.

### DNMT1 expression correlated with DACH1 methylation and expression in colorectal cancer

**FIGURE 3:**
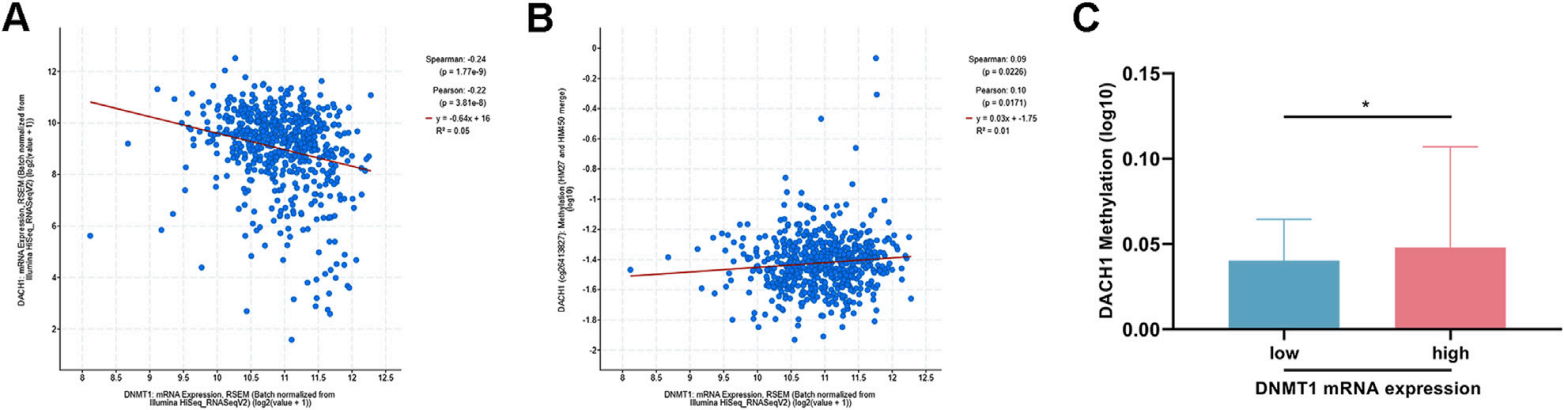
Relationship of DNMT1 expression with DACH1 expression and methylation in colorectal cancer. Correlation analysis of DNMT1 and **(A)** DACH1 mRNA expression as well as **(B)** DACH1 promoter methylation levels using TCGA database. **(C)** DACH1 methylation levels in patients divided based on median DNMT1 levels. Data are presented as mean ± SEM. *p < 0.05.

**FIGURE 4:**
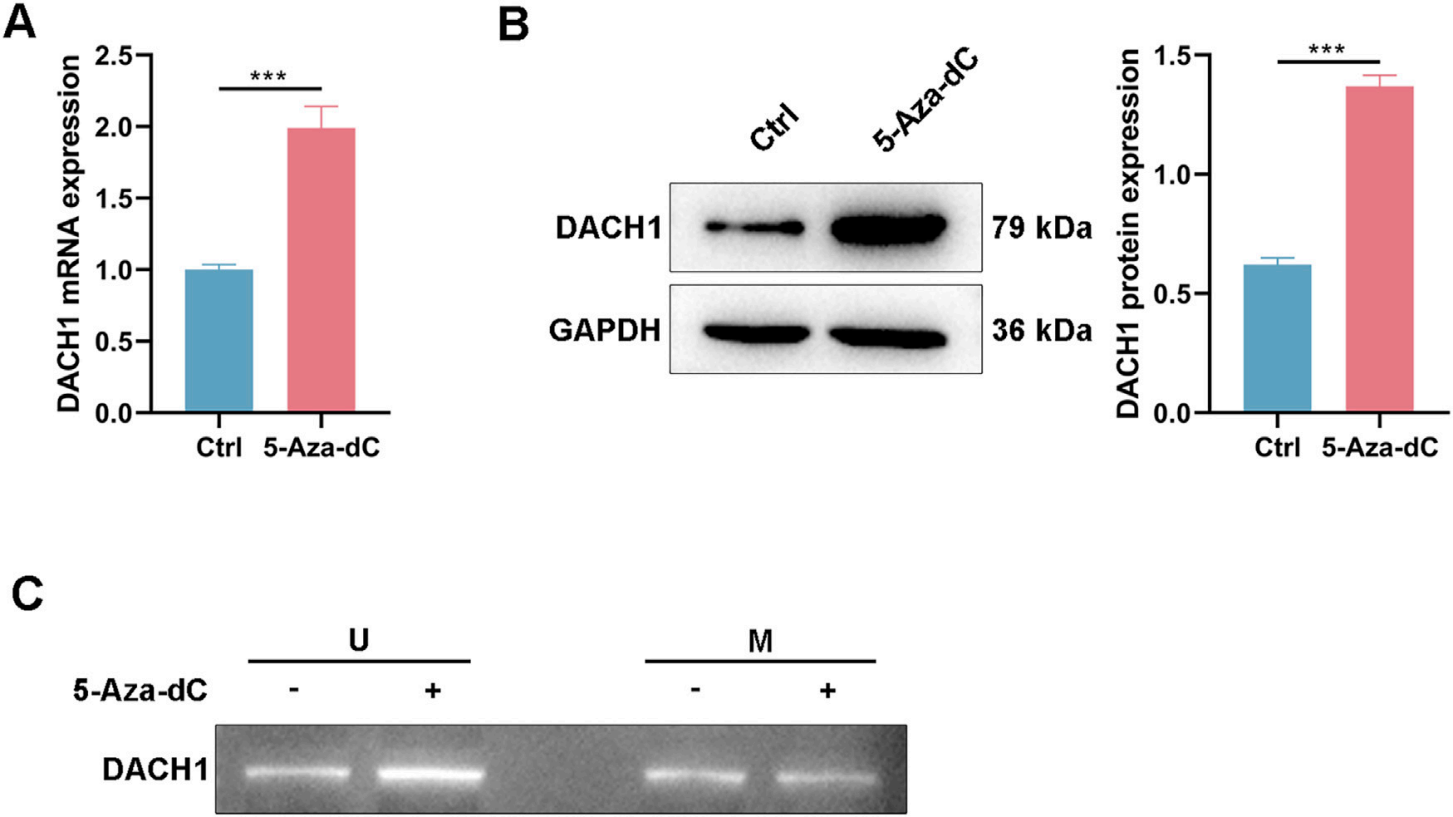
DACH1 expression was regulated by DNMT1-mediated methylation *in vitro*. After being exposed to a DNMT1 inhibitor (5-Aza-dC), HCT116 cells were collected for analyzing the **(A)** mRNA, **(B)** protein, and **(C)** methylation levels of DACH1. Data are presented as mean ± SEM. ***p < 0.001.

DNMT1 is a key regulator of DNA methylation. Analysis of TCGA data of CRC revealed a significant negative correlation between DNMT1 and DACH1 mRNA expression levels (Figure 3A). Furthermore, DNMT1 expression was positively correlated with DACH1 promoter methylation levels (Figure 3B). Moreover, patients stratified into two groups based on the median DNMT1 expression level showed consistent results, with higher DNMT1 expression associated with significantly increased DACH1 methylation, while lower DNMT1 expression correlated with reduced methylation (Figure 3C). Furthermore, *in vitro* experiment using a DNMT1 inhibitor (5-Aza-dC) was performed, which indicated that the mRNA, protein, as well as methylation levels of DACH1 in HCT116 cells were significantly reduced after treating with 5-Aza-dC (Figures 4A–C).

The clinical significance of DACH1 and DNMT1 expression levels was also evaluated in CRC patients. No significant differences in either marker was observed between older (≥60 years) and younger patients or between males and females. However, tumor differentiation, lymphatic metastasis, and tumor stage were significantly associated with DACH1 and DNMT1 expression. Poorly differentiated tumors exhibited lower DACH1 expression and higher DNMT1 expression compared to moderately differentiated tumors. Similarly, tumors with lymphatic metastasis showed significantly reduced DACH1 expression and elevated DNMT1 expression compared to non-metastatic cases. Advanced-stage tumors (DUCKS stages C and D) were characterized by markedly lower DACH1 expression and higher DNMT1 expression compared to early-stage tumors (Table 1). These findings suggested that DACH1 downregulation and DNMT1 upregulation might be closely associated with tumor progression in CRC. The reciprocal relationship between DNMT1 expression and DACH1 methylation and expression underscored the epigenetic regulation of DACH1 by DNMT1.

**TABLE 1:** Comparison of DNMT1 and DACH1 expression in patients with different clinicopathological parameters.

Clinical parameters	Number of cases	DACH1 expression			DNMT1 expression		
		Low	High	*p* value	Low	High	*p* value
Age				0.525			0.525
<60	22	10	12		10	12	
≥60	18	10	8		10	8	
Gender				0.752			0.342
Male	21	11	10		12	9	
Female	19	9	10		8	11	
Differentiation degree				0.003^*^			0.022^*^
High	15	2	13		11	4	
Medium-Low	25	18	7		9	16	
Lymphatic metastasis				0.003^*^			0.022^*^
Yes	15	12	3		4	11	
No	25	8	17		16	9	
DUCKS stage				0.001^*^			0.038^*^
A, B	25	9	19		17	11	
C, D	15	11	1		3	9	

Note: p^*^<0.05 indicates statistic differences.

### DNMT1 expression progressively increased in colorectal cancer progression and negatively correlated with DACH1 expression

**FIGURE 5:**
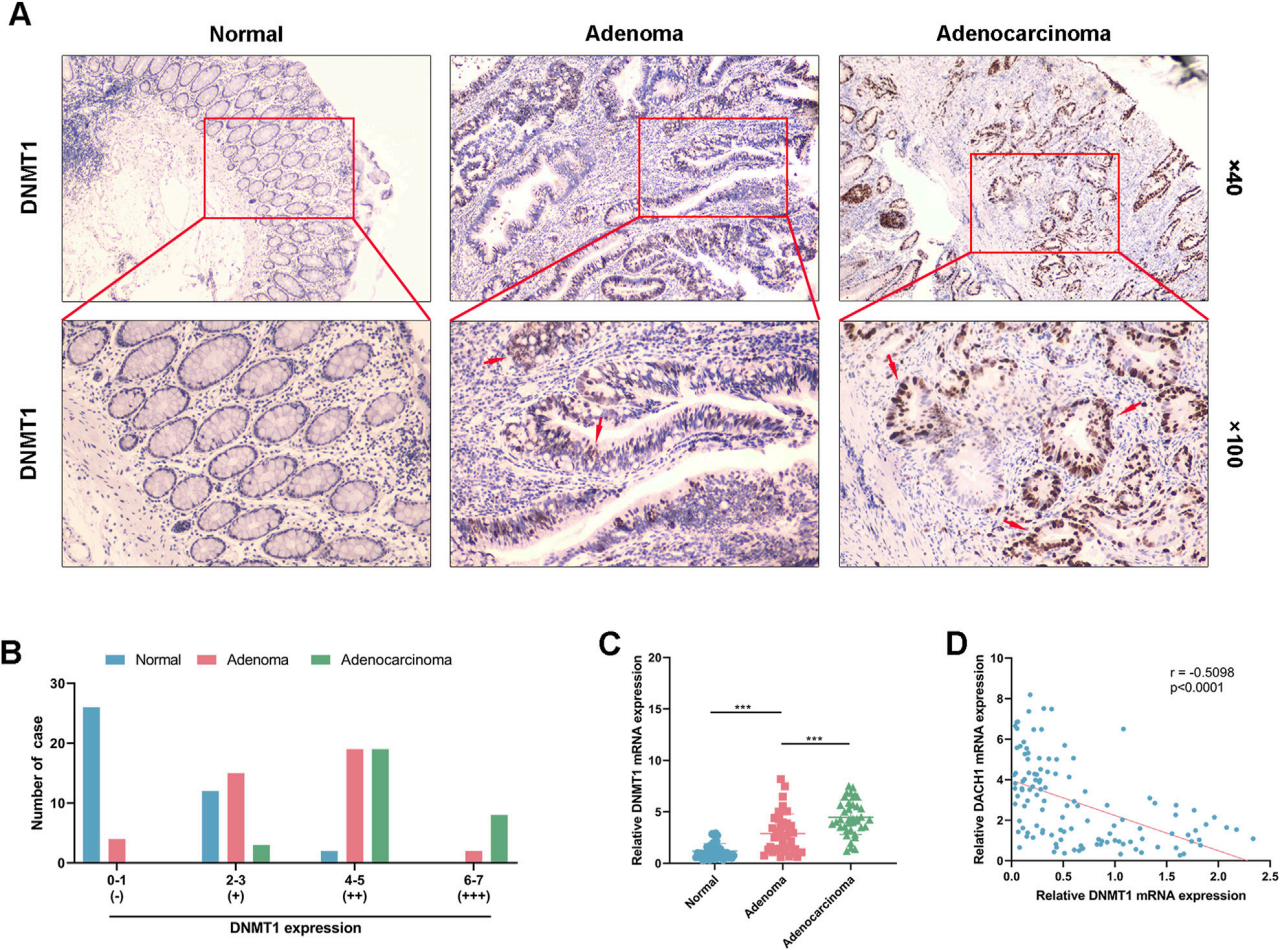
DNMT1 expression in colorectal cancer progression. **(A)** Immunohistochemical analysis of DNMT1 protein expression in normal colorectal mucosa, adenoma, and adenocarcinoma tissues of patients (magnifications used were 100 × and 400 ×) and **(B)** semi-quantitative scoring of staining defined as negative (−), weak (+), moderate (++), and high (+++) levels. **(C)** Quantitative PCR analysis of DNMT1 mRNA levels from normal mucosa to adenoma and adenocarcinoma tissues. **(D)** Correlation analysis of DNMT1 and DACH1 mRNA expression conducted by Pearson’s correlation analysis. Data are presented as mean ± SEM. ***p < 0.001.

To further investigate the role of DNMT1 in CRC progression, its expression was analyzed and compared across normal colorectal mucosa, adenoma, and adenocarcinoma tissues using IHC and PCR. IHC results demonstrated a progressive increase in DNMT1 protein expression along the normal-adenoma-adenocarcinoma sequence (Figure 5A). Semi-quantitative scoring of DNMT1 staining revealed that the proportion of cases with negative staining (−) was highest in normal mucosa and significantly reduced in adenoma and adenocarcinoma tissues, with no cases observed in adenocarcinoma. Weakly positive stained cases (+) demonstrated progressively decreased trend followed normal, adenoma and adenocarcinoma. Moderately positive staining (++) was highest in adenoma, followed by adenocarcinoma, and lowest in normal tissues. Moreover, strongly positive staining (+++) was absent in normal tissues, but significantly more frequent in adenocarcinoma compared to adenoma (Figure 5B). PCR analysis of DNMT1 mRNA expression further confirmed a stepwise increase in expression levels from normal mucosa to adenoma and adenocarcinoma tissues (Figure 5C). Notably, DNMT1 mRNA expression was found to be negatively correlated with DACH1 mRNA expression (r = −0.5098, p < 0.0001) (Figure 5D), as previously observed in Figure 1C. Overall, DNMT1 expression exhibited a progressive increase at both the protein and mRNA levels during the transition from normal mucosa to adenoma and adenocarcinoma. The inverse correlation between DNMT1 and DACH1 expression highlighted the potential role of DNMT1 in silencing tumor suppressor genes DACH1 through epigenetic mechanisms.

### DACH1 and DNMT1 proteins had opposite expression patterns across normal, adenoma, and carcinoma tissues from the same patient

**FIGURE 6:**
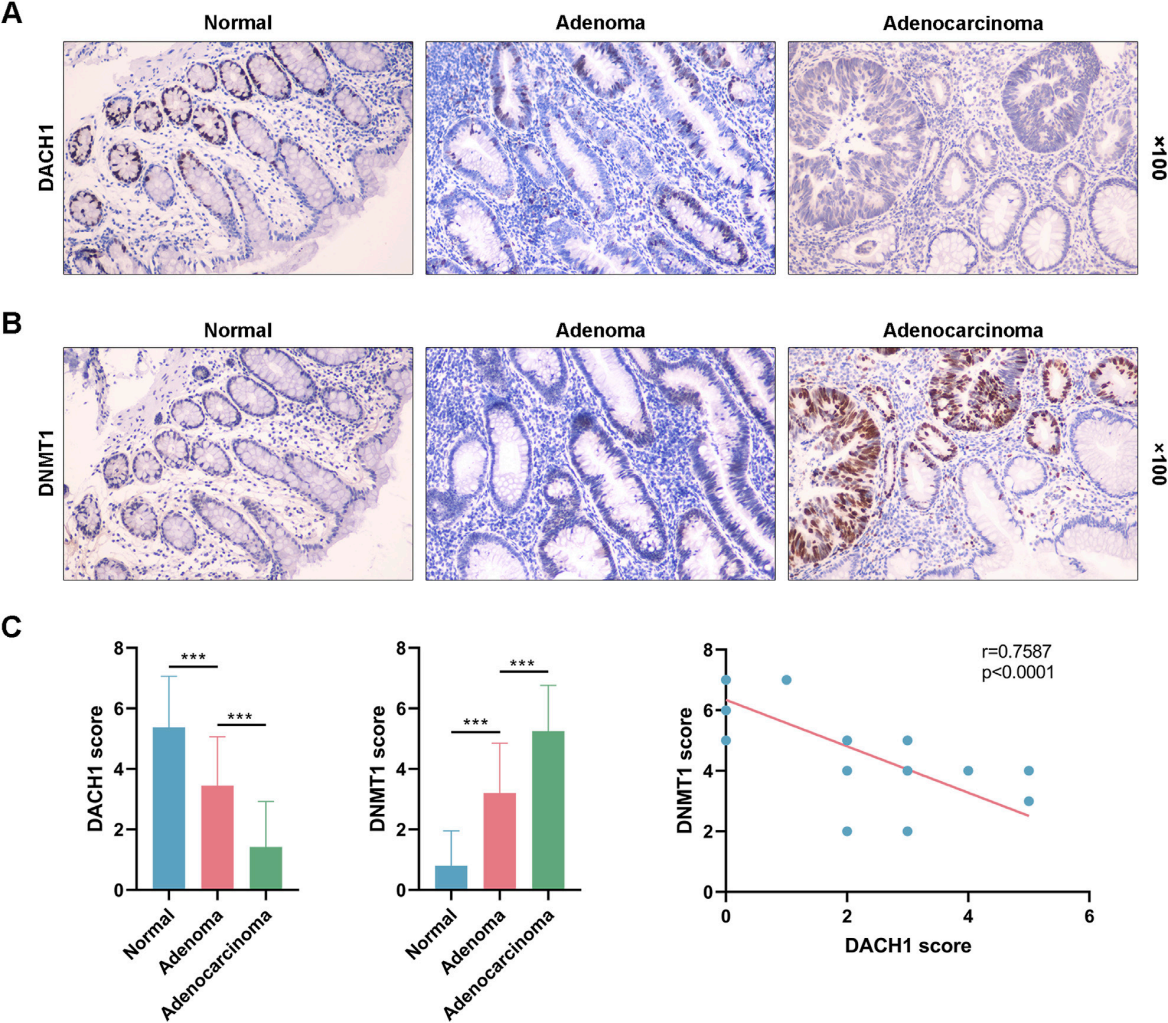
DACH1 and DNMT1 protein expression patterns across normal, adenoma, and carcinoma tissues from the same patient. Three types of tissues were collected from a patient to evaluate the protein levels of **(A)** DACH1 and **(B)** DNMT1 by immunohistochemical analysis. The magnifications used were 100 ×. **(C)** The DACH1 and DNMT1 expressions were scored, and a correlation analysis was performed between them.

Normal mucosa, adenoma, and carcinoma tissues obtained from the same patient were used to detect the expression of DACH1 and DNMT1 during CRC progression by IHC. DACH1 protein expression was found to decrease progressively from normal to adenoma to carcinoma tissues. Normal mucosa exhibited the highest levels of DACH1 expression, which was markedly reduced in adenoma and further diminished in carcinoma tissues (Figure 6A). In contrast, DNMT1 protein expression demonstrated an opposite trend, with the lowest expression in normal mucosa, moderate expression in adenoma, and the highest levels in carcinoma tissues (Figure 6B). These expression trends across tissues from the same patient supported the notion that DACH1 downregulation and DNMT1 upregulation play critical roles in CRC progression. Finally, the correlation of expression levels between DACH1 and DNMT1 was plotted, which revealed a potential relationship between DACH1 and DNMT1 in CRC (Figure 6C).

## Discussion

The incidence and mortality rates of CRC are steadily increasing, posing a significant threat to human health [18]. The progression of CRC typically follows the normal mucosa-adenoma-carcinoma sequence, investigating the molecular mechanisms underlying which and identifying potential targets for intervention are essential for preventing CRC. In this study, we combined bioinformatics analyses with clinical sample validation to elucidate the expression trends of DACH1 levels, DACH1 promoter methylation, and DNMT1 levels across different stages of CRC progression and our findings revealed that DNMT1-mediated epigenetic silencing of DACH1 plays a pivotal role in driving CRC initiation and progression.

DACH1 was recognized as a tumor suppressor gene in our study, supported by a significant decrease in DACH1 protein and mRNA expression from normal mucosa to adenomas and adenocarcinomas. This was aligning with previous findings linking the loss of DACH1 expression to tumor development and poor prognosis [6, 19]. The silencing of tumor suppressor genes like DACH1 in CRC may result from various mechanisms, including epigenetic regulation such as promoter DNA methylation, aberrant histone modifications, and the involvement of non-coding RNAs [20]. Our findings demonstrated that the progressive decrease in DACH1 expression during CRC progression corresponds with a concurrent increase in promoter methylation, confirming that DNA methylation might be one of a critical mechanism driving DACH1 silencing in this context. This mechanism is not unique to CRC, as CpG island hypermethylation has similarly been shown to silence DACH1 in other cancer types. For instance, the DACH1 methylation rate was illustrated higher levels in non-small cell lung cancer compared to adjacent normal lung tissues and demethylation treatment contributed to decreased migration and invasion of cancer cells [21]. This emphasized the broader significance of this epigenetic regulation in tumor progression.

DNA methylation is regulated by DNMTs, among which DNMT1 is a primary enzyme responsible for maintaining DNA methylation patterns. DNMT1 recognizes hemimethylated CpG dinucleotides with the assistance of the auxiliary factor UHRF1. Using S-adenosylmethionine as a methyl donor, DNMT1 adds methyl groups to cytosines on the newly synthesized DNA strand, preserving the methylation state during DNA replication. [22, 23]. Aberrant overexpression of DNMT1 has been widely reported across various cancers and is frequently associated with hypermethylation and silencing of tumor suppressor gene promoters [24, 25]. For example, DNMT1 was overexpressed in pancreatic ductal adenocarcinoma tissues, with a opposite trend in normal pancreatic tissues and silencing of it demethylated Bax gene promoter, which demonstrated increased mRNA expression [26]. Consistent with this, our present study revealed a significant positive correlation between DNMT1 expression and DACH1 promoter methylation, accompanied by a negative correlation between DNMT1 expression and DACH1 transcription, which also in line with a progressively elevated trend across the CRC progression sequence.

Machine learning based on database analysis has previously predicted numerous critical genes and regulatory axes involved in CRC progression, providing valuable insights into potential therapeutic targets for cancer treatment. Sardari et al. [27] utilized a multivariate machine learning strategy across multiple independent datasets in a recent study and identified genes such as TNS4, SLC7A5, and SCD, which were demonstrated to be associated with CRC. However, these predictions require validation through clinical evidence. Existing studies on gene regulatory mechanisms in CRC primarily focus on database-based predictions or animal models [28–31], with relatively few integrating with clinical sample validation. The strength of our study lies in addressing this gap by combining bioinformatics analyses with multi-layered verification using clinical samples, systematically confirming the expression patterns and regulatory interactions of DACH1 and DNMT1 at critical stages of CRC progression. Specifically, based on the TCGA database, we analyzed RNA sequencing data and DNA methylation profiles from CRC patients. This revealed a significant negative correlation between DACH1 expression and its promoter methylation as well as DNMT1 expression, suggested that the probability of DNMT1 to mediate DACH1 transcriptional silencing by promoting hypermethylation of its promoter. To further confirmation, we collected clinical samples representing normal mucosa, adenomas, and adenocarcinomas tissues. A progressive decrease in DACH1 expression from normal mucosa to adenoma to adenocarcinoma were observed, while DNMT1 expression showed a corresponding increase, demonstrating an inverse relationship in both protein and mRNA levels. BSP analysis further revealed the hypermethylation of the DACH1 promoter, supporting the hypothesis obtained *in silico*. Additionally, paired analysis of samples from individual patients contributed to eliminating inter-patient variability and clearly demonstrated dynamic changes in molecular expression during the normal mucosa-adenoma-carcinoma sequence within the same patient. The consistent findings with the overall trends observed in the larger cohort reinforced the opposing expression patterns and cooperative regulatory mechanism between DACH1 and DNMT1 during CRC progression.

In addition to elucidating the regulatory axis between DACH1 and DNMT1, our findings demonstrated a strong association between the expression levels of these two factors and clinicopathological features of CRC. Patients with poorly differentiated tumors, advanced stages, or lymphatic metastasis exhibited significantly lower DACH1 expression and higher DNMT1 expression, underscoring the potential of aberrant expression of DACH1 and DNMT1 as molecular biomarkers for the early diagnosis and prognostic evaluation of CRC. From a treatment perspective, DNMT1 inhibitors such as 5-azacytidine have been shown to be able to reverse SPARC promoter hypermethylation and thereby improving therapeutic efficacy [32]. This highlights the potential of epigenetic therapies targeting DNMT1 as a benefit for CRC patients.

The present study also has certain limitations. We primarily relied on the analysis of tissue samples, and while the findings suggest an association between DNMT1 overexpression, DACH1 promoter hypermethylation, and gene silencing, we did not further evaluate these mechanistic links through functional experiments like DNMT1 inhibition or knockdown. Additionally, although we integrated TCGA data with analyses of 120 clinical samples, the relatively small sample size may limit the generalizability of the results. The clinical relevance of DNMT1 and DACH1 requires further validation in larger, multicenter cohorts and preclinical therapeutic studies to provide a more comprehensive understanding of the DNMT1-DACH1 regulatory axis and its potential clinical applications in CRC patients.

Our study revealed the critical role of DNMT1-mediated DACH1 methylation in CRC progression through multi-layered analyses integrating bioinformatics and clinical samples. The progressive increase in DNMT1 expression drives the epigenetic silencing of DACH1 via promoter methylation, facilitating the transition from adenoma to carcinoma. This process is closely associated with clinical tumor differentiation, lymph node metastasis, and DUKES staging. Our findings provided significant evidence supporting the potential of the DNMT1-DACH1 regulatory axis as a biomarker and therapeutic target for CRC patients.

## Data Availability

The original contributions presented in the study are included in the article/Supplementary Material, further inquiries can be directed to the corresponding author.

## Footnotes

1
https://www.cbioportal.org/

2
http://www.urogene.org/cgi-bin/methprimer/methprimer.cgi

3
http://www.urogene.org/methprimer/
